# What guidance exists to support remote consultations in sexual and reproductive health services? A review of the policy and practice literature

**DOI:** 10.1136/sextrans-2025-056519

**Published:** 2025-08-25

**Authors:** Charlotte Spurway, Iestyn Williams, Christian Bohm, Oluseyi Cyril Ayinde, Fiona Burns, Jo Gibbs, Jo Josh, Helen Munro, Danielle Solomon, Melvina Woode Owusu, Jonathan D C Ross, Louise J Jackson

**Affiliations:** 1 Department of Applied Health Sciences, College of Medicine and Health, University of Birmingham, Birmingham, UK; 2 Health Services Management Centre, University of Birmingham, Birmingham, UK; 3 Whittall Street Clinic, University Hospitals Birmingham NHS Foundation Trust, Birmingham, UK; 4 Institute for Global Health, University College London, London, UK; 5 PPIE Lead, London, UK; 6 Hywel Dda University Health Board, Carmarthen, UK

**Keywords:** SEXUAL HEALTH, REPRODUCTIVE HEALTH, Telemedicine

## Abstract

**Abstract:**

**Introduction:**

The use of remote consultations, such as appointments via telephone, video, online or text in sexual and reproductive health services (SRHS) across the UK, has expanded in recent years. This review synthesises grey literature from different organisations to identify current practice and guidance for remote consultations.

**Methods:**

We searched for a range of grey literature document types, including unpublished reports, evaluations, published standards, guidance, blogs and opinion pieces. The searches were conducted between March 2023 and July 2024 using Google, as well as the Healthcare Management Information Consortium database and preidentified organisational websites (eg, the British Association for Sexual Health and HIV, the Faculty of Sexual and Reproductive Healthcare). Data extracted included terminology, challenges to implementation and linked guidance and equity considerations in the use of remote consultations in SRHS. Narrative synthesis was used to analyse findings.

**Results:**

The available guidance on implementing and delivering remote SRHS is modest in scope and volume and draws on a sparse evidence base. Existing guidance recommends the use of safeguarding assessments and checklists to support pathways from remote into in-person care. While remote consultations were seen as potentially enhancing equity, challenges included differences in technology access and digital literacy. Equity-related guidance included prioritising disadvantaged groups for in-person appointments and flexible care pathways.

**Discussion and conclusions:**

The grey literature highlights the potential of remote SRHS to improve access and equity while also identifying risks in implementation and outcomes. There is an ongoing requirement for detailed, evidence-informed guidance that incorporates service user perspectives.

**KEY MESSAGES**

Remote consultations have been integrated into many sexual and reproductive health services (SRHS), alongside in-person appointments. Existing evidence suggests that remote consultations can present challenges that affect equity, access and the quality of service delivery. This synthesis reveals a notable lack of guidance tailored specifically to the unique challenges and characteristics of SRHS, with limited insight into delivering SRHS in the postpandemic era. Future research should prioritise developing flexible, proactive strategies to integrate in-person and remote consultations. Further evidence is needed to inform best practices and equity-focused guidance.

## Introduction

 The COVID-19 pandemic led to a rapid reduction in in-person consultations and an expansion in the volume and scope of ‘remote’ consultations, which involve the use of telecommunications or digital technology as a substitute for in-person contact between clinicians and service users.^1^ For sexual and reproductive health services (SRHS), this involved the use of remote consultations to diagnose, treat and manage sexually transmitted infections (STIs) and to provide contraception and abortion care. This review synthesises grey literature on remote consultations in SRHS and identifies current best practices and guidance for implementation. It also assesses how equity and intersectionality (ie, how aspects such as race, gender, class and sexuality interact to shape experiences) are addressed in the existing guidance for SRHS. It complements a separate systematic review of peer-reviewed evidence on remote consultations delivered in SRHS.^2^ This review is relevant to SRHS providers, policymakers and researchers seeking to optimise services and address inequities.

## Background

In SRHS, the use of remote consultations represents a major change to service delivery, but we lack a full understanding of the impact on service quality and equity. By offering alternatives such as telephone, video, online or text consultations, remote methods provide new ways to access care. While there are several potential benefits to remote consultations, such as increased convenience and the potential to reduce costs,^3 4^ the optimal configurations of remote and in-person services remain unclear.

SRHS face unique challenges in service delivery due to the stigma surrounding STIs, contraception and abortion care, which can create significant barriers to access. Vulnerable groups, including those from lower sociodemographic backgrounds, ethnic minorities and people with mental health conditions, often experience poorer sexual and reproductive health and face greater barriers to accessing care.^5 6^ There is concern that remote consultations might exacerbate these disparities by introducing technological barriers and reducing choice.^7^ Digital exclusion is strongly associated with lower educational attainment and social disadvantage, and this raises the risk of poorer outcomes for individuals already at increased risk of poor sexual and reproductive health.^8^

The COVID-19 pandemic disrupted SRHS, with reduced in-person access, service interruptions and appointment delays contributing to unmet need.^9^ Remote consultations were introduced as a strategy to address these gaps. In this context, the limited guidance available on implementation and best practices makes it unclear whether services are equipped to meet the complex needs of users. Furthermore, SRHS play an important role in identifying and supporting survivors of sexual and domestic violence. During periods of change, such as the COVID-19 pandemic, shifts in service delivery models may reduce opportunities for disclosure and access to support.^10^ However, clear guidance on implementation, including safeguarding protocols and delivery of care, is also limited.

While guidance exists to aid use of remote consultations in general healthcare,^11 12^ there is a lack of comprehensive guidance tailored to SRHS. This creates uncertainty, especially in conditions of resource constraint. In England, substantial reductions in SRHS budgets have created pressures to reduce costs, while services in Wales have reportedly struggled to meet demand.^13^ This is echoed by findings from the Terrence Higgins Trust^14^, which found significant pressures and resource constraints on sexual health services in England, Scotland and Wales, affecting the availability of in-person appointments.^14^ To understand how remote consultations are being used by SRHS, we reviewed the grey literature to identify any existing guidance on the use of remote consultations in SRHS and synthesised the key messages relating to both implementation and equity considerations.

## Methods

Given our focus on implementation and equity within UK services, we drew on characteristics protected under UK antidiscrimination law (eg, age, disability, sex, gender, marital status, race, religion, sexual orientation). The National Health Service (NHS) in England ‘Core20PLUS5’ framework was also used to orient us to potentially disadvantaged populations (eg, people experiencing homelessness, vulnerable migrants, people with learning disabilities and autistic individuals, and rural/coastal communities).^15^ The Core20PLUS5 framework also identifies priority clinical areas, although these do not include SRHS.

The overall approach to searching was iterative and exploratory, in keeping with the diffuse nature of the grey literature sought. We searched for: unpublished reports, evaluations, published standards, guidance and guidelines, blogs and opinion pieces, news articles, policy documentation (including policy briefs) and non-systematic literature reviews. Our primary focus was literature relating to SRHS; however, we also explored documents providing generic guidance on remote consultations in the UK NHS. Documents were included if they were recent (ie, 2018–2024), published by credible sources such as UK government departments, professional and healthcare organisations, academic institutions or established healthcare charities, and published in English. While no geographical limits were set for inclusion, the searches primarily focused on UK-based sources (see below). Where multiple documents from the same organisational body with little variation between them were identified, one of these was randomly selected for inclusion.

There were three strands to the search strategy:

- We conducted an internet search using Google. Google was chosen as a search tool due to its ability to cover a wide range of sources, including those not indexed in specific databases, and to provide up-to-date, globally relevant results. This was updated several times between March 2023 and July 2024, combining terms on remote consultations/remote services with terms on sexual and reproductive health (online supplemental file 1, appendix 1). We adopted a broad definition of remote consultations, which included literature on telemedicine, telehealth, online SRHS and digital SRHS.
- We conducted searches of the Healthcare Management Information Consortium (HMIC) database,^16^ which formally includes grey literature related to health and social care management. We searched for general guidance documents on remote consultations as well as documents on equity and/or cost-effectiveness (online supplemental file 1, appendix 2).
- We conducted a targeted search of the websites of professional organisations (mostly UK based) working in the field of sexual and reproductive healthcare, including the British Association for Sexual Health and HIV (BASHH) and the Faculty of Sexual and Reproductive Healthcare (FSRH) (online supplemental file 1, appendix 3).

In addition to the three search strands, some documents were identified by the coauthors as potentially relevant for inclusion. We devised a bespoke data extraction tool combining descriptive information (date, author, document type, intended audience, scope/aims, setting) (Appendix 4, (online supplemental file 1, appendix 4) and recording the following substantive data:

- Links to current/existing policies on remote consultations and any reasons for introducing remote consultations (eg, COVID-19, resource constraints).
- Any existing data or evidence cited in support of any guidance.
- Good practice guidance and implications for practice.
- References to equity/protected characteristics and how these may combine to create unique experiences of disadvantage (intersectionality).

Data were extracted independently by two of the authors (CS and IW) who then discussed and resolved any differences with the other authors. Data synthesis followed a narrative approach in keeping with the textual nature of the included documents.^17^ Two of the authors (CS and IW) led on the synthesis, which involved comparison across the extracted dataset. From this, we constructed narrative summaries against each of the main research aims and questions and selected verbatim quotations to illustrate the main findings. The following structure was used to report findings: definitions and types of remote consultations; relevant policies, existing evidence; general challenges and guidance; and equity-related challenges and guidance. Identified themes were then discussed and refined with the full author team.^18^

## Results

Our search identified limited literature offering specific, practical guidance on the remote delivery of SRHS. Table 1 includes information about the grey literature documents included in this review. Nearly all of the documents included in the review were identified through Google searches, with a small number found via searches of organisation websites and contributions from coauthors. No documents from HMIC were identified for inclusion in the review. Although the majority were located through Google searches, there was considerable overlap between the sources. For example, some documents were found both through Google and on organisation websites. This indicates that the search strategy was effective in capturing the key documents.

**Table 1:** Grey literature documents and their characteristics

Authors/organisation (country/area)	Title	Type of document	Specialist area	Summary	Source*
Abacan^24^	Telehealth for sexual health among adolescents and young adults	Blog	Sexual health	The blog highlights digital access and privacy barriers to equitable telehealth for low-income, minority service users.	Google
Academy of Medical Royal Colleges^38^	Telemedicine and early termination of pregnancy	Statement	Abortion	Council of the Academy of Medical Royal Colleges statement in support of the permanent implementation of telemedicine for EMA.	Google
Båge and Datta^23^	Report on access to contraception in Europe during the COVID-19 pandemic	Report	Contraception	This document describes five main barriers and enabling factors related to access to contraception in Europe during COVID-19. The document is not explicitly about remote consultations in SRHS but does mention digital tools and telemedicine.	Google
BASHH^30^	BASHH position statement on multiplex testing platforms and antibiotic regimens	Statement	STIs	Position statement about dangers of online providers in the UK delivering STI testing and treatment. The document states that online services are not providing first-line treatments for confirmed STIs as recommended by national clinical guidelines.	Google
BHIVA^20^	Guidance for virtual consultations for people with HIV	Guidance	HIV	The document provides guidance on virtual consultations for HIV around patient choice over modality, equity of access, clinic resources and infrastructure, privacy, confidentiality and safeguarding, and HIV-specific considerations.	Google
Brook Young People^32^	Brook response to ending provision of telemedicine abortion	Press release	Abortion	Brook statement showing support for continued use of remote consultations for abortion services.	Google
Cameron *et al*^29^	Evaluation of telemedicine early medical abortion at home in Scotland	Report	Abortion	The report recommends continuing home use of mifepristone, improving information and access, enhancing care options and increasing support for staff to strengthen telemedicine EMA at home in Scotland.	Google
FSRH and BASHH^19^	Standards for online and remote providers of sexual and reproductive health services	Standards	Sexual and reproductive health	The document provides standards to be used as a benchmark for all online providers of SRHS, and as a tool for the commissioning of services. The document provides five standards mirrored by those from the Care Quality Commission enquiry for online providers of primary healthcare.	Google
FSRH and BASHH^42^	Expansion of online services and new standards for online SRH providers	Blog	Sexual and reproductive health	Blog post about the FSRH and BASHH joint standards for online providers of sexual and reproductive health.	FSRH website
FSRH and BASHH^41^	Teletriage for SRH services in response to COVID-19	Report	Sexual and reproductive health	This document outlines key considerations for implementing or expanding teletriage for remote SRHS. It includes example models, minimum skill requirements, essential checklists, and guidance on remote support and call handling.	Google
FSRH and RCOG^33^	Medical bodies oppose ending telemedicine abortion service	Statement	Abortion	The document provides a statement in support of EMA at home. It provides evidence from existing research to support keeping telemedicine abortion services.	Google
Gotsadze^40^	Impact of COVID-19 on SRH services in Georgia	Report	Sexual and reproductive health	The report presents findings from a qualitative study which aimed to determine the impact of COVID-19 on access to essential SRHS. It notes the lack of clear guidance on remote consultations, argues remote abortion should be continued post pandemic.	Google
Honeyman *et al*^43^	Digital technology and health inequalities: a scoping review	Scoping review	Health inequalities	This review examines how equality can be promoted or risks mitigated in the design and use of digital technologies.	Identified by co-authors
Ilett^28^	Redesigning SRH service in Scotland during the pandemic	Blog	Sexual and reproductive health	This blog post provides an overview of the results of a qualitative survey carried out in summer 2020 during COVID-19. Participants involved in the study were FSRH committee members.	FSRH website
Karsten and West^27^	Telehealth apps expand access for reproductive health care	Commentary	Reproductive health	This commentary advocates for the use of telehealth apps to enhance access to contraception and sexual health services. It also addresses key challenges associated with these platforms, including legal and regulatory issues, safety concerns and digital security.	Google
Llywodraeth Cymru^35^	The quality statement for women and girls’ health	Report	Women’s health	This document outlines expectations for Welsh health boards to ensure quality care for women and girls, including adopting a blended approach with digital and virtual consultations.	Google
Mostaghim^47^	How telemedical early medical abortion could protect women and pregnant people	Blog	Abortion	The blog post argues that telemedical EMA allows for greater and safer access to reproductive choices for vulnerable women and pregnant people.	Google
Nadarzynski *et al*^21^	Do HIV patients find remote services acceptable?	Poster	HIV	This document presents findings from a survey on digital services for people with HIV. It highlights concerns around security and confidentiality and shows that while remote consultations are moderately acceptable overall, acceptance is higher among those with greater confidence in using online platforms.	Google
National Coalition of STD Directors^26^	Adolescent sexual health telemedicine services during COVID-19	Report	Sexual health	This report from the US provides a toolkit for delivering telemedicine sexual health services to adolescents. It provides guidance on how services should be delivered and identifies the challenges such as privacy and confidentiality.	Google
NICE^39^	Abortion care: accessibility and sustainability of services	Guidance	Abortion	This document reviews existing evidence in relation to the accessibility and sustainability of abortion services. The document provides evidence to support the use of telemedicine in EMA, although it does not provide any specific guidance on how this should be done.	Google
O’Sullivan^31^	How London boroughs are embracing digital technologies for sexual health	Blog	Sexual health	The document refers to online sexual health services, which includes mail-order STI kits via a website. The blog states that if follow-up care is needed, there is a dedicated team of health advisors.	Google
Predmore and Rollinson^22^	Telemedicine abortion? It’s not as easy as it sounds	Commentary	Abortion	The commentary provides some options for policymakers and providers to support use, such as exploring audio-only telemedicine, increasing access to safe and private spaces and raising awareness about telemedicine options.	Google
RCOG^36^	Best practice in telemedicine for abortion care	Report	Abortion	The document provides guidance on the best practices for abortion care by telemedicine using existing evidence. The guidance covers a broad range of stages including developing trust, safeguarding, determining pregnancy duration and taking medical histories and contraception discussions.	Google
RCPCH^34^	Safeguarding guidance for under-18s accessing abortion services	News article	Abortion	The document provides an overview of guidance created by RCPCH about safeguarding guidance for children and young people accessing EMA services.	Google
Sturgiss *et al*^25^	COVID-19 and access to SRH for young people: international overview	Literature review	Sexual and reproductive health	This review aims to inform policymakers and practitioners about sexual and reproductive healthcare access for young people during COVID-19.	Google

* We identified other literature during our search which has not been included in the final literature review. These additional documents can be found in online supplemental file 1, appendix 5.

BASHH, British Association for Sexual Health and HIV; BHIVA, British HIV Association; EMA, early medical abortion; FSRH, Faculty of Sexual and Reproductive Healthcare; NICE, National Institute for Health and Care Excellence; RCOG, Royal College of Obstetricians and Gynaecologists; RCPCH, Royal College of Paediatrics and Child Health; SRHS, sexual and reproductive health services; STIs, sexually transmitted infections.

The most common document types were reports, position statements and blogs published by a range of organisations. Most documents focused on remote consultations, abortion care and SRHS in general, with fewer documents focusing explicitly on contraception, STI testing and/or HIV screening. Many of the documents did not directly cite any evidence in support of the claims made, beyond references to previous guidance and policy documents. Where published evidence was cited, this was largely restricted to surveys of service providers and/or discussions with local stakeholders, with more rigorous evaluations missing. This need for further evidence is recognised in some of the included documents. For example, FSRH-BASHH note ‘current research around SRH provision through online services is still in its infancy, though growing, and we hope that any developing evidence will be used to shape the online services of the future’.^19^ Similarly, British HIV Association (BHIVA) guidance on virtual consultations for people with HIV 2021 notes ‘there is currently a limited evidence base to guide provision of virtual HIV care and it is important that HIV service models are adapted as further evidence becomes available’.^20^

We included a small number of documents that, although not focussing on SRHS, provided guidance on remote services, either in general or in other service areas (eg, general practice, community pharmacy, physiotherapy). These documents outline a range of factors influencing remote consultations and present recommendations which are summarised in table 2.

**Table 2:** Generic guidance and recommendations on remote consultations in the delivery of healthcare

Recommendations	Sources
Codesign remote consultation systems with staff and patients and conduct workforce planning and skills gap analysis before proceeding.	^11 12 48^
Provide choice and flexibility over modalities in consultation with patients and use in-person modes for physical examinations when necessary.	^11 12 49 50^
Assess patient capability to use digital services; offer in-person options or support to vulnerable patients.	^12^
Develop policies around consent, confidentiality, capacity and safeguarding.	^51 52^
Develop patient selection criteria, risk stratification, triage pathways and/or standard operating procedures.	^11 12 48 49 51^
Provide clear and accessible information to patients about appointment types, booking and confidentiality.	^12 48 50^
Invest in digital infrastructure and use encrypted, confidential video calling methods.	^11 12 48 53^
Support staff with pressures of remote consulting through training, guidance and peer learning.	^11 12 48 50 51^
Evaluate and monitor the impact of remote consultations.	^12 48^
Conduct equality and health impact assessments before remote consultations and use a population health management approach.	^48^
Use asynchronous communication (emails, messaging) for specific situations, but avoid for complex or sensitive consultations.	^50^

### Implementing remote delivery

Security and confidentiality of digital services were commonly noted as challenges with remote consultations,^19 21 22^ especially where requirements exist for proof of identity, indicated by FSRH-BASHH guidance.^19^ Privacy issues, such as data protection and ensuring patients have access to a private area for remote consultations, were also reported.^22^,^26^ Additionally, the necessity for stable internet access and connection, along with the availability of a smartphone or device, was recognised as presenting a significant challenge to accessing services as not everyone has access to these.^23 27^ Other risks linked to remote consultations included loss of face-to-face interaction due to the absence of non-verbal cues and reduced opportunities for spontaneous discussions with patients.^20 28 29^ Safeguarding challenges were also noted in the BHIVA^20^ guidance on virtual consultations, which highlighted that at the start of virtual consultations, patient identity, safety and location and contact details should be verified.

Documents acknowledged that digital service modes can limit the scope of services offered, for example, precluding provision of long-acting reversible contraception or STI testing during remote consultations for early medical abortion.^29^ FSRH-BASHH guidance^19^ states that pathways should be in place to facilitate in-person appointments if physical examinations are required. A lack of institutional support for remote consultations, such as limited funding and technology for implementation, also creates challenges in service delivery, and there are additional risks associated with unregulated private health services delivering remote consultations in SRHS.^30^

The FSRH-BASHH standards mandate that online providers of SRHS adhere to five key areas: safety; effectiveness; treating people with kindness, respect and compassion; responsiveness; and good governance and leadership.^19^ Much of the included literature supports the permanent integration of remote consultations in SRHS and abortion care, noting that online services can provide additional ways to engage with care.^31^ Specifically, ‘no test’ models of abortion care, which use ultrasound scans only when clinically necessary, are argued to be safe, effective and acceptable to women.^32 33^ However, children and young people should be offered in-person consultations for early medical abortion to help identify and address any potential safeguarding concerns.^34^ Notably, the included literature did not address the cost implications or cost-effectiveness of implementing remote services in SRHS, leaving an important gap in understanding.

Developing standards for remote consultations, such as essential checklists for staff, was also identified as important.^19^ Further recommendations include ensuring that SRHS offering remote consultations provide clear and accessible information about the available options, such as through frequently asked questions (FAQ) sheets and infographics, and promote the availability of remote abortion care where this option exists.^22^ Services should be person centred, based on individual needs and preferences, with remote consultations offered as an option alongside in-person services.^35^ Technologies used in remote consultations should be codesigned with patients to improve their experience and outcomes.^35^ To ensure inclusivity of online services, providers are advised to continually review and improve digital tools and give appropriate training to staff on using digital services for appointments.^20 23^ Furthermore, to ensure data safety, telehealth apps should use encryption and multifactor authentication.^27^

Guidance on safeguarding for service providers included the adoption of telephone or video call safeguarding risk assessments or ‘safe words’ to ensure providers can adequately identify people at risk.^34 36 37^ The FSRH-BASHH standards^19^ include specific safeguarding guidance for identifying potentially vulnerable young people, those with mental health conditions, adults lacking capacity and those with complex medical histories/polypharmacy. Elsewhere it is recommended that: clinical staff involved in remote consultations are trained to level 3 safeguarding; sexually active young people (16–18 years) undergo a child sexual exploitation risk assessment; assessment is made of recreational drug and alcohol abuse and domestic violence, and each service has a designated safeguarding lead. Results from these assessments should redirect patients to in-person services when appropriate.

### Equity considerations

Several included documents reference UK Equality, Diversity and Inclusion related legislation such as the 2010 Equality Act and the 2005 Mental Capacity Act. Where the question of equity was directly addressed, this was most often with the intention of asserting the equity gains afforded by remote consultation, for example, by improving access for those living in rural settings, those with disabilities and women in abusive relationships.^36 38^

A smaller proportion of the literature included reflections on how remote consultation might pose access challenges and risks. For example, this might present in the form of lack of access to technology (such as smartphones) or the skills required for their use. This was identified as an issue for older age groups and ‘underserved populations’.^24 26 27^ Other documents noted that lack of privacy at home can be a barrier to telemedicine,^23^ and this may apply disproportionately to underserved populations,^24^ or vulnerable groups such as women at risk or gender-based violence.^23^

Despite recognition of these challenges and risks, few of the included documents provided specific guidance on ensuring equity in remote consultation. From those that did, the following recommendations were put forward:

- Being prepared to prioritise disadvantaged groups for in-person appointments.^28^ However, there is a lack of clarity with regards to the circumstances that would warrant reversion to an in-person consultation and how to implement this change.
- Where possible, seeking to provide resources to support the use of technology and the use of the internet.^24^
- Introducing flexibility into pathways to remote consultation and ensuring any outreach activities are accessible to the most vulnerable groups.^39^
- Making information available in all languages, including sign language.^40^

A small number of documents provided the bulk of the available equity-related guidance.^19 33 41 42^ Many of the remaining documents were brief news articles, blog posts or organisational statements, with limited scope to provide detailed guidance. One document argued for equality impact assessments to be carried out on remote consultation policies alongside risk assessments covering drug and alcohol abuse, domestic violence and disability.^42^ They further recommend that user interfaces promote diversity according to protected characteristics, and for language support, for those with English not as a first language, to be made available.

Equity-related guidance put forward for remote consultation and abortion^36^ included that information be made available in different languages and formats, providers should use ‘value neutral, unbiased language’ and may require a trusted adult or social worker in consultations with patients who are ‘very young or vulnerable’.^19 42 43^ Training should be provided to practitioners in identifying intimate partner violence and reproductive coercion in a remote consultation setting. None of the included documents directly considered how the intersection of factors might produce specific barriers, risks or challenges.

## Discussion and conclusion

This is the first synthesis of existing guidance for remote delivery of SRHS in the UK. While our primary focus was on the UK, we found that some documents from outside the UK described similar challenges and recommendations. Notably, generic guidance on remote consultations in NHS healthcare highlights several factors that are also relevant to SRHS, such as the importance of codesigning systems with staff and patients and the need for staff training and rapport building. However, a key difference lies in the emphasis within SRHS guidance on understanding how factors such as age, gender, sexual orientation and socioeconomic status intersect to shape patients’ experiences. Given these overlaps and differences, there is a clear need to further adapt generic NHS guidance to meet the specific demands of SRHS.

At the same time, searches of the grey literature suggest that while remote delivery offers the potential to improve access, it also poses risks related to both implementation and outcomes. The current literature includes some feedback and guidance to those implementing and delivering remote SRHS, but these are constrained by a lack of robust evidence, which aligns with broader concerns about evidence gaps in telemedicine in other areas of the NHS.^44^ Much of the existing evidence focused on how organisational and institutional policies can help to mitigate risks to equity of access.

Overall, there is a lack of clarity about when in-person appointments are preferable, for example, relating to age or language skills. While it is generally recognised that individuals under 18 and those likely to face exclusion from digital services should be offered in-person appointments, the option of allowing patients to choose their preferred appointment type is rarely addressed. As a result, there is limited guidance on how to effectively integrate in-person and remote consultations within the same service. Future work might focus on more flexible and proactive strategies to integrate in-person and remote consultations, ensuring that new service models maximise equity and address the diverse needs of all patients. Existing guidance rarely captures the full complexity of remote SRHS delivery, particularly in relation to service-specific needs and evolving policy contexts. This gap is further compounded by the fact that a significant proportion of the documentation relates to the COVID-19 period, with limited focus on how consultations should be provided in the post-COVID-19 era. This issue is especially evident in the context of legal changes surrounding telemedicine abortion in England, Scotland and Wales,^45^ which highlights the importance of clear guidance on managing remote consultations.

In the wider literature, there is some guidance around organisational and institutional policies for ensuring equity of access. However, much more focus on this area would be beneficial, given the differential prevalence of STIs and unplanned pregnancies across social groups. In particular, we found a lack of consensus on when and how to revert to in-person consultations, as well as on the optimal provision of hybrid models. This gap was impacted by a lack of attention to wider social forces that drive social disadvantage, and that intersectionality was not directly addressed. Additionally, there was little evidence in the guidance documents regarding the cost-effectiveness and/or financial implications of different models of care, highlighting a significant gap in knowledge.

There are several strengths associated with this study. First, an extensive and iterative search was conducted, drawing on documents from key stakeholder groups including clinicians, some patient representatives and those involved in policy development and implementation. Second, an inclusive approach was adopted for the incorporation of literature from key organisations in the UK in different formats. Nonetheless, we acknowledge that there are limitations. Although our search was extensive, it is possible that some guidance documents might have been missed, as grey literature search and synthesis is inherently challenging. In particular, our focus at the national level likely meant that local guidance documents were missed. Second, while our search focused on SRHS, some relevant materials from other health areas might have provided useful insights, for example, consultations for mental health services.

There is a need for increased focus on the SRHS context for guidance on remote services, and particularly in relation to remote consultations. The guidance documents themselves acknowledge the paucity of evidence, highlighting the need for further research on how to implement remote consultations effectively in SRHS.^19^ This research should also explore how remote services can complement and support in-person consultations. Further evidence is needed, in particular, to inform guidance around equity and best practice. This should consider how the needs of diverse populations can be met and potential barriers understood and overcome. It is important to involve service users in planning and evaluating remote services to ensure they can meet the needs of different groups.^46^ Several of the included documents refer to involving service users and public engagement in the design of service delivery, to ensure that services can be accessible.^19 23^ Furthermore, intersectionality is an important consideration that requires further exploration and guidance in relation to SRHS.

## Supplementary material

10.1136/sextrans-2025-056519 online supplemental file 1

## Data Availability

All data relevant to this study are provided within the article or included as supplemental materials.
