# Supplementary material for: What guidance exists to support remote consultations in sexual and reproductive health services? A review of the policy and practice literature
Source: Sex Transm Infect. 2025 Aug 25;101(8):e056519. doi: 10.1136/sextrans-2025-056519 (PMC12703314; doi:10.1136/sextrans-2025-056519)
Supplement: online supplemental file 1 [file sextrans-101-8-s001.docx]

**SUPPLEMENTAL MATERIALS**

**Appendix 1: Search terms used for google search**

**Search terms used:**

“remote consultations” [and] “sexual health”

“remote consultations” [and] “reproductive health”

“telemedicine” [and] “sexual health”

“telemedicine” [and] “reproductive health”

“Online” [and] “sexual health services”

“remote services” [and] “sexual health”

“remote services” [and] “reproductive health”

“remote consultations” [and] contraception

“remote consultations” [and] abortion

Telemedical abortion

“remote consultations” [and] antenatal

“remote consultations” [and] perinatal

“remote consultations” [and] chlamydia

“remote consultations [and] “family planning”

“telephone consultations” [and] “sexual health”

“telephone consultations” [and] “reproductive health”

“video consultations” [and] “sexual health”

“video consultations” [and] “reproductive health”

“online consultations” [and] “sexual health”

“online consultations” [and] “reproductive health”

“digital consultations” [and] “sexual health”

“digital consultations” [and] “reproductive health”

NB Each combination of terms was searched on Google in the UK, initially as an unrestricted search, then as a search restricted to results from the past year only. Included in the results were items published

**Appendix 2: HMIC Health Management Information Consortium <1979 to May 2024> database search strategy**

Search Strategy:
1  ((remote$ or online or virtual or telephone or phone or video or digital) adj3 (consultation$ or service$ or advi$)).mp. (1774)
2  ("telephone call$" or "telephone advice" or "telephone advisory").ti,ab. (414)
3  telemedicine.mp. or exp telemedicine/ or telecare/ (2104)
4  telehealth.mp. or exp telehealth/ (885)
5  exp telephone consultations/ or exp telephone services/ or telephone advice/ (1293)
6  1 or 2 or 3 or 4 or 5 (4557)
7  (guide$ or guidance or implement$ or introduc$ or evaluat$ or standard$ or barrier$).ti. or Guidelines/ or Standards/ or Implementation/ or Evaluation/ or Service planning/ (52802)
8  (equit$ or inequalit$ or equal$ or unequal$).ti. or Health inequalities/ (10016)
9  (cost or effective$).ti. (8527)
10  7 or 8 or 9 (68818)
11  6 and 10 (1093)
12  exp Prescribing/ (3680)
13  prescribing.ti. (1913)
14  (prescri$ or drug$ or medication$ or pharmac$).ti. (14257)
15  general practice.mp. or exp General practice/ (15556)
16  primary care.mp. or exp primary care/ (34464)
17  12 or 13 or 14 or 15 or 16 (51542)
18  11 and 17 (259)

**Appendix 3: Organisations for targeted search**

**Organisations:**

British Association for Sexual Health and HIV

British HIV Association

Faculty of Sexual and Reproductive Healthcare

NICE

WHO

NHS England

Public Health Wales

Public Health Scotland

UK HSA

OHID

Terrence Higgins Trust

Family Planning Association

NHS Digital

**Appendix 4: Data extraction tool**

CONNECT study grey literature review data extraction form

1. **Document characteristics**

a) Title:

b) Date published/accessed:

c) Authors/sponsor:

d) Document type:

Unpublished report

Audit/evaluation

Standards/guidance

Blogs/opinion piece

Policy brief

Other policy document

Unstructured review

Other (specify)

Add detail on document type:

|  |
| --- |

Comments about document:

|  |
| --- |

1. **Scope and audience**

a) Definition of remote consultations

|  |
| --- |

b) Intended audience

NHS

UK Local government

Policy

Patients/users

Other country/system

Other (specify)

|  |
| --- |

c) Service user/population groups covered

develop checklist as we go through documents

Other (specify)

|  |
| --- |

d) Relevant policies presented or referenced

|  |
| --- |

e) Evidence cited in support of the document (including any previously unpublished data)

|  |
| --- |

1. **Insights into remote consultations**

a) Challenges of remote consultation (general) (e.g., implementation, costs, safety)

|  |
| --- |

b) Guidance on remote consultation (general)

|  |
| --- |

c) Equity challenges in remote consultation

|  |
| --- |

d) Guidance on equity in remote consultation

|  |
| --- |

e) Checklist of marginalised groups/sources of inequity addressed

Ethnic minority groups  specify: ………………………………………………………………………….

Other protected characteristics  specify: ………………………………………………………………………….

P/w learning disability

P/e homelessness

P/e drug & alcohol dependence

Vulnerable migrants

Gypsy, Roma and Traveller

Sex workers

P. in contact with justice system

Victims of modern slavery

Other  specify: ………………………………………………………………………….

f) References to intersectionality

Yes  No

|  |
| --- |

1. **Other relevant information and notes**

|  |
| --- |

**Appendix 5: Additional documents**

1. All-Party Parliamentary Group on Sexual and Reproductive Health. Women's Lives, Women's Rights: Strengthening Access to Contraception Beyond the Pandemic. London: All-Party Parliamentary Group; 2020.
2. British Medical Association Cymru Wales. Consultation response: Termination of pregnancy arrangements in Wales. Cardiff: British Medical Association; 2021.
3. Boso Pérez M, et al. What were patients’ experiences of trying to access sexual and reproductive health services during the early months of the COVID-19? BMJ Sexual & Reproductive Health Blog. 2022 Jun 20.
4. British Society of Abortion Care Providers. BSACP Position Statement: Remote Consultations. Coventry: BSACP; 2020.
5. British Society of Abortion Care Providers, Royal College of Obstetricians and Gynaecologists. Telemedical abortion care: Safeguarding young people. London: RCOG; 2021.
6. Faculty of Sexual and Reproductive Healthcare. FSRH consultation response: FSRH responds to Welsh Government’s consultation on the home use of abortion pills. London: FSRH; 2021.
7. Faculty of Sexual and Reproductive Healthcare. The FSRH Hatfield Vision: A Framework to Improve Women and Girls’ Reproductive Health Outcomes. London: FSRH; 2022.
8. Horgan G, Gray AM, Morgan L. Developing Integrated Sexual & Reproductive Health Services in Northern Ireland. Belfast: ARK; 2019.
9. Moore J, Baraitser P. Understanding the cost of quality within an online sexual health service. In: Curtis L, Burns A, editors. Unit Costs of Health & Social Care 2019. Canterbury: Personal Social Services Research Unit, University of Kent; 2019. p. 13–20.
10. Pilkington V, Serrecchia C, Edwards A. Are phone clinics the future? Evaluating patient experiences of telemedicine in a regional integrated sexual health service. Sex Transm Infect. 2022;98(Suppl 1):A65.1.
11. Romanis EC, Parsons JA. Abortion: under-18s pushed towards in-person appointments but evidence shows remote consultations can be better option. The Conversation. 2023 Jan 27.
12. Rough E. Early Medical Abortion at Home During and After the Pandemic. London: House of Commons Library; 2022.
13. Royal College of Paediatrics and Child Health. RCPCH statement on early medical abortion by telemedicine. London: RCPCH; 2022.
14. Weigel G, Frederiksen B, Ranji U, Salganicoff A. Telemedicine in Sexual and Reproductive Health. San Francisco: Henry J. Kaiser Family Foundation; 2019 Nov.
15. World Health Organization. WHO issues consolidated guide to running effective telemedicine services. Geneva: WHO; 2022.
16. World Health Organization. Self-management of medical abortion via telemedicine in Germany. Geneva: WHO; 2023.
